# Tenascin C Has a Modest Protective Effect on Acute Lung Pathology during Methicillin-Resistant Staphylococcus aureus-Induced Pneumonia in Mice

**DOI:** 10.1128/spectrum.00207-21

**Published:** 2021-07-28

**Authors:** Mariska T. Meijer, Alex F. de Vos, Hessel Peters Sengers, Brendon P. Scicluna, Joris J. Roelofs, Chérine Abou Fayçal, Fabrice Uhel, Gertraud Orend, Tom van der Poll

**Affiliations:** a Center for Experimental and Molecular Medicine, Amsterdam University Medical Centers, University of Amsterdam, Amsterdam, the Netherlands; b Amsterdam Institute for Infection and Immunity, Amsterdam University Medical Centers, University of Amsterdam, Amsterdam, the Netherlands; c Clinical Epidemiology Biostatistics and Bioinformatics, Amsterdam University Medical Centers, University of Amsterdam, Amsterdam, the Netherlands; d Department of Pathology, Amsterdam University Medical Centers, University of Amsterdam, Amsterdam, the Netherlands; e The Tumor Microenvironment Laboratory, INSERM UMR_S 1109, Université Strasbourg, Faculté de Médecine, Hopital Civil, Institut d'Hématologie et d'Immunologie, Fédération de Médecine Translationnelle de Strasbourg (FMTS), Strasbourg, France; f Division of Infectious Diseases, Amsterdam University Medical Centers, University of Amsterdam, Amsterdam, the Netherlands; Emory University School of Medicine

**Keywords:** tenascin C, *Staphylococcus aureus*, methicillin-resistant *Staphylococcus aureus*, pneumonia, alarmins, innate immunity, immune system, mice, Gram-positive bacterial infections

## Abstract

Tenascin C (TNC) is an extracellular matrix protein with immunomodulatory properties that plays a major role during tissue injury and repair. TNC levels are increased in patients with pneumonia and pneumosepsis, and they are associated with worse outcomes. Methicillin-resistant Staphylococcus aureus (MRSA) is a Gram-positive bacterium that is a major causative pathogen in nosocomial pneumonia and a rising cause of community-acquired pneumonia. To study the role of TNC during MRSA-induced pneumonia, TNC sufficient (TNC^+/+^) and TNC-deficient (TNC^−/−^) mice were infected with MRSA via the airways and euthanized after 6, 24, and 48 h for analysis. Pulmonary transcription of TNC peaked at 6 h, while immunohistochemistry revealed higher protein levels at later time points. Although TNC deficiency was not associated with changes in bacterial clearance, TNC^−/−^ mice showed increased levels of TNF-α and IL-6 in bronchoalveolar lavage fluid during the acute phase of infection when compared with TNC^+/+^ mice. In addition, TNC^−/−^ mice showed more severe pulmonary pathology at 6, but not at 24 or 48 h, after infection. Together, these data suggest that TNC plays a moderate protective role against tissue pathology during the acute inflammatory phase, but not during the bacterial clearance phase, of MRSA-induced pneumonia. These results argue against an important role of TNC on disease outcome during MRSA-induced pneumonia.

**IMPORTANCE** Recently, the immunomodulatory properties of TNC have drawn substantial interest. However, to date most studies made use of sterile models of inflammation. In this study, we examine the pathobiology of MRSA-induced pneumonia in a model of TNC-sufficient and TNC-deficient mice. We have studied the immune response and tissue pathology both during the initial insult and also during the resolution phase. We demonstrate that MRSA-induced pneumonia upregulates pulmonary TNC expression at the mRNA and protein levels. However, the immunomodulatory role of TNC during bacterial pneumonia is distinct from models of sterile inflammation, indicating that the function of TNC is context dependent. Contrary to previous descriptions of TNC as a proinflammatory mediator, TNC-deficient mice seem to suffer from enhanced tissue pathology during the acute phase of infection. Nonetheless, besides its role during the acute phase response, TNC does not seem to play a major role in disease outcome during MRSA-induced pneumonia.

## INTRODUCTION

Tenascin C (TNC) is an extracellular matrix (ECM) protein that plays a major role during embryonic development. In adult tissue, TNC is expressed at low levels but becomes upregulated at sites of mechanical stress or tissue injury (1, 2). Recently, TNC has garnered attention as a putative damage-associated molecular pattern molecule with immunomodulatory properties (3, 4). TNC promotes the production of proinflammatory cytokines in response to bacterial products in multiple cell types, including macrophages and dendritic cells (5). In addition, TNC has been shown to stimulate inflammatory processes in a variety of inflammatory disease models (3, 6–10), as well as in models of pulmonary inflammation such as ovalbumin-induced asthma (11) and bleomycin-induced lung fibrosis (12, 13). In patients, increased plasma TNC concentrations have been reported during pneumonia and pneumosepsis (14, 15), and elevated TNC levels have been reported in both serum and bronchoalveolar lavage fluid (BALF) of COVID-19 patients (16). Finally, TNC plays a crucial role during tissue repair mechanisms (17).

Methicillin-resistant Staphylococcus aureus (MRSA) is a Gram-positive bacterium that is a major causative pathogen in nosocomial pneumonia and a rising cause of community-acquired pneumonia, although its prevalence varies between countries and continents (18–20). MRSA is able to produce a wide array of toxins and can cause severe pneumonia with extensive tissue damage, potentially resulting in a necrotizing infection (21). Moreover, MRSA is able to secrete immunomodulatory proteins that aid in evading the host immune system but also lead to more severe inflammation and tissue damage (22).

Since TNC is upregulated at sites of inflammation and tissue damage, we hypothesized that its expression would increase during MRSA-induced pneumonia and that it would be involved in the host inflammatory response during this infection. In addition, TNC could participate in antibacterial defense, considering its effects on professional phagocytes crucial for clearance of MRSA (3–5, 22) and its potential to interact directly with bacteria (23). Therefore, in the current study we sought to determine the role of TNC in inflammation and bacterial clearance during MRSA-induced pneumonia. For this purpose, we performed *in vivo* experiments inducing pneumonia in TNC deficient (TNC^−/−^) and wild-type (TNC^+/+^) mice by infection with MRSA via the airways.

## RESULTS

### TNC is upregulated in lungs but does not impact bacterial clearance during MRSA-induced pneumonia.

**FIG 1:**
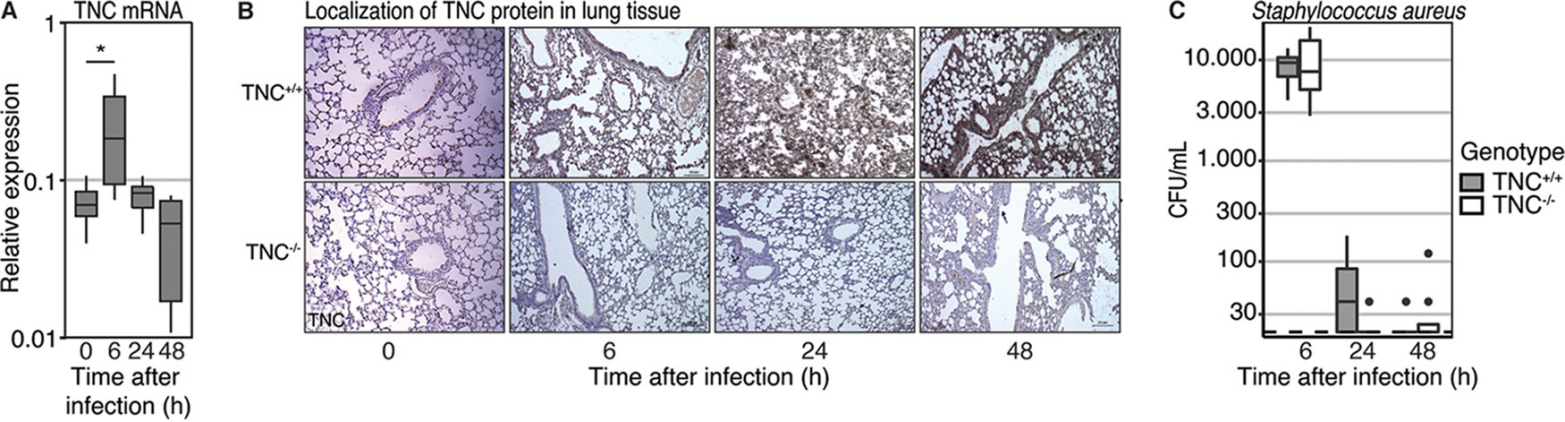
Tenascin C expression is induced during a transient pulmonary infection with methicillin-resistant Staphylococcus aureus. (A) Mice were intranasally infected with MRSA. RNA was extracted from the lung homogenate of mice sacrificed before infection and at 6, 24, or 48 h thereafter. TNC mRNA expression was determined in the tissue of TNC^+/+^ mice through qPCR and normalized to HPRT1 mRNA content. (B) Immunohistochemistry staining of tenascin C in pulmonary tissue during infection. Pictures are representative of 5 independent samples. (C) BALF was collected upon sacrificing mice at 6, 24, and 48 h after infection. The number of CFU in BALF was determined through overnight culture on blood agar plates. Data are shown as a Tukey boxplot. TNC mRNA levels were compared to baseline using unpaired *t* tests with Holm’s adjustment. Bacterial outgrowth between TNC^+/+^ and TNC^−/−^ mice after 6 h was compared using an unpaired *t* test. Later time points were limited to descriptive statistics without hypothesis testing. The dashed line represents the lower limit of detection. **, *P* < 0.01 (compared to naive).

Infection with MRSA via the airways resulted in enhanced TNC mRNA levels in lungs of TNC^+/+^ mice at 6 h (*P* = 0.01 versus naive mice; Fig. 1A). Induction of TNC mRNA was transient, as it returned to baseline levels after 24 h (*P* = 0.80) and remained so at 48 h (*P* = 0.75). As expected, TNC mRNA was not induced in TNC^−/−^ mice. Immunohistochemistry (IHC) staining showed accumulation of TNC protein in the lung ECM of TNC^+/+^ mice throughout the duration of the model, while TNC^−/−^ tissue remained negative (Fig. 1B). TNC protein was not localized in a specific site in the lung, but rather distributed throughout the ECM compartment.

Bacterial loads were similar in BALF of TNC^+/+^ and TNC^−/−^ mice obtained 6 h after infection and MRSA was similarly cleared from the airways in both mouse strains thereafter (Fig. 1C). Bacterial dissemination to blood and distal organs was limited in both groups (Table S1 in the supplemental material).

### TNC plays a limited role in monocyte/alveolar macrophage responses and cytokine release in the bronchoalveolar space during MRSA-induced pneumonia.

**FIG 2:**
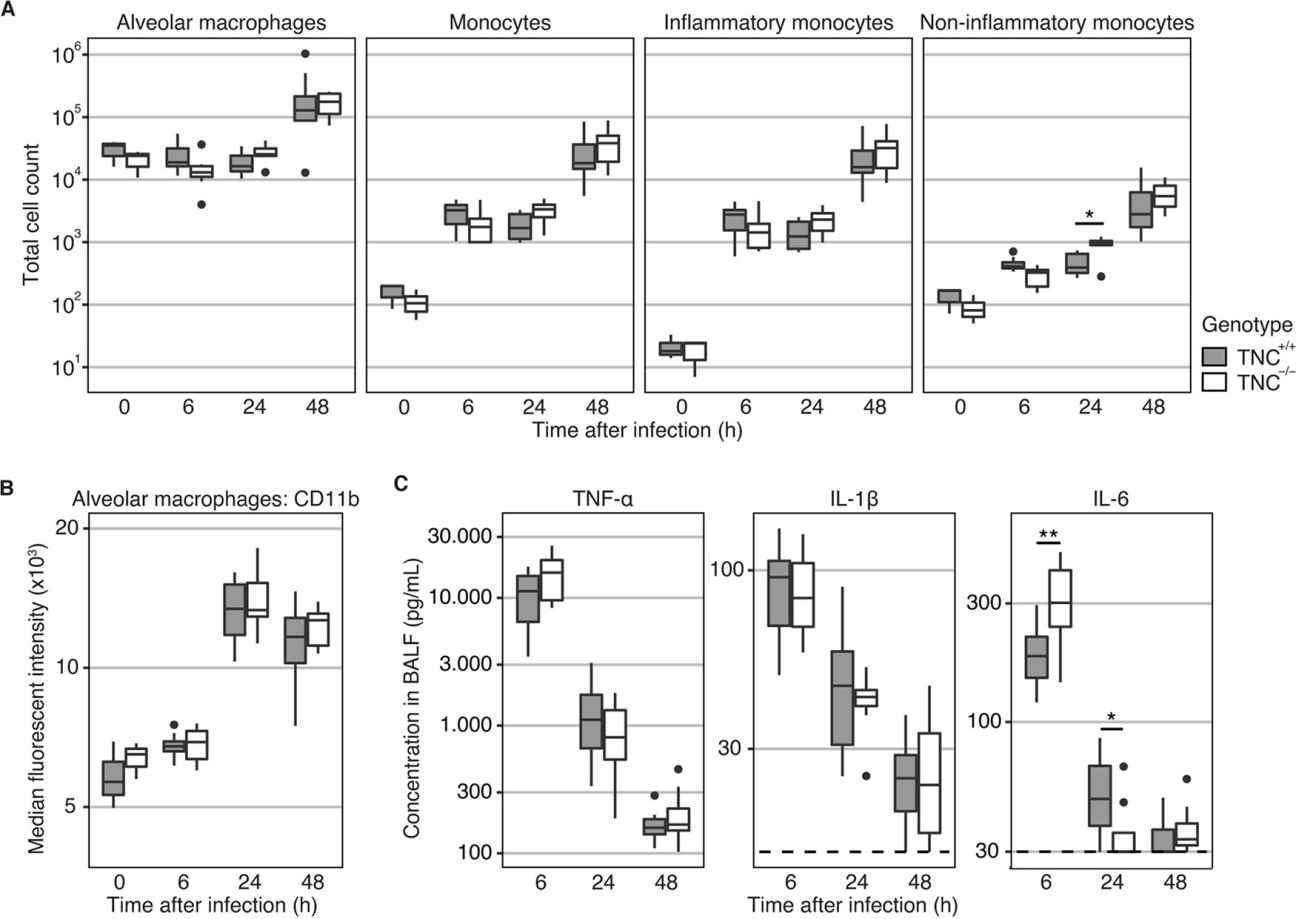
Tenascin C is not required for the recruitment and activation of monocytes during pulmonary infection with methicillin-resistant Staphylococcus aureus. Mice were intranasally infected with MRSA. Before infection and at 6, 24, and 48 h thereafter, mice were sacrificed and unilateral BALF was collected. (A) Total numbers of alveolar macrophages, monocytes, inflammatory monocytes, and noninflammatory monocytes were determined through fluorescence-activated cell sorting (FAC) analysis. (B) The expression of CD11b on alveolar macrophages was measured through the median fluorescence intensity during FACS analysis. (C) Cytokine levels in BALF were determined through ELISA. Data are shown as Tukey boxplots. Statistical analysis was performed using linear models. Dashed lines indicate the lower limit of quantification. *, *P* < 0.05; **, *P* < 0.01.

TNC is known to regulate the innate immune response of macrophages and monocytes (3–5). To study the recruitment of these cells during MRSA-induced pneumonia, we measured total cell numbers of alveolar macrophages (CD45^+^CD11c^+^Siglec-F^+^) and monocytes (CD45^+^CD11c^−^Siglec-F^−^Ly6-G^−^Ly6-C^−^CD11b^+^) in BALF before and at 6, 24, and 48 h after infection (Fig. 2A). Within the monocyte subsets, we quantified inflammatory monocytes (Ly6C^hi^) as well as noninflammatory monocytes (Ly6C^lo^). All of these mononuclear cell subsets were recruited to the alveolar space during the infection and remained present until 48 h after infection. However, this occurred independently of the presence of TNC (*P* < 0.01 for time and *P* > 0.1 for genotype in all subsets). Only noninflammatory monocyte infiltration was altered (interaction: *P* = 0.020), with significantly more infiltration in the TNC^−/−^ group after 24 h (*P* = 0.028), but not the other time points. Likely, the change in this minor subset it driven by the expansion of monocytes as a whole. To assess alveolar macrophage activation, we quantified CD11b expression in this subset (Fig. 2B). While the CD11b expression increased significantly over time (*P* < 0.01), there were no differences between the TNC^+/+^ and TNC^−/−^ mice across time (*P* = 0.14).

In addition to markers of cell activation, we measured BALF levels of tumor necrosis factor alpha (TNF-α), interleukin 1β (IL-1β), and IL-6 (Fig. 2C). While TNF-α and IL-1β were both produced during the early stages of infection, neither was dependent on TNC (*P* = 0.74 and *P* = 0.75, respectively). IL-6 levels in BALF, on the other hand, did vary by genotype (*P* = 0.59 for genotype but *P* = 0.005 for genotype over time). More specifically, IL-6 was increased in TNC^−/−^ mice compared to TNC^+/+^ mice (*P* = 0.009) at 6 h after infection. Conversely, after 24 h, IL-6 levels had decreased below the TNC^+/+^ levels (*P* = 0.04), while IL-6 levels were low in both genotypes at 48 h after infection (*P* = 0.64).

### The absence of TNC does not alter neutrophil influx into the lung and bronchoalveolar space.

**FIG 3:**
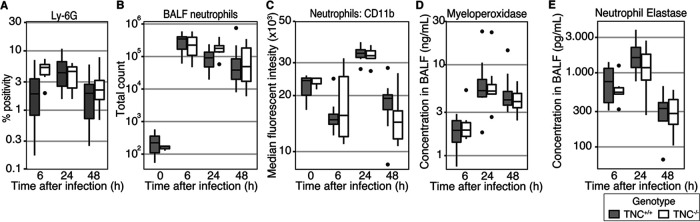
Tenascin C plays a limited role in neutrophil recruitment and activation during methicillin-resistant Staphylococcus aureus*-*induced pneumonia. Mice were intranasally infected with MRSA. Before infection and at 6, 24, and 48 h thereafter, mice were sacrificed. Unilateral BALF and contralateral lung tissue was collected. (A) Tissue was fixed, embedded in paraffin, and stained for Ly-6G. Ly-6G positivity is expressed as a percentage of total tissue surface. (B) Total neutrophil content of BALF was analyzed through FACS. (C) Neutrophil CD11b surface expression levels were assessed through the median fluorescence intensity during FACS analysis. (D and E) BALF myeloperoxidase (D) and neutrophil elastase (E) levels were determined through ELISA. Data are shown as Tukey boxplots. Statistical analysis was performed using linear models.

While most literature focuses on the role of TNC in the regulation of monocyte and macrophage immunity, neutrophils play a major role in the clearance of MRSA (22). Therefore, we investigated the influx and activation of neutrophils. First of all, we studied neutrophil influx in lung tissue through quantification of IHC Ly-6G staining (Fig. 3A). Although not significant, there was a trend toward an increased percentage of Ly-6G-positive lung tissue in the TNC^−/−^ mice (*P* = 0.07 for genotype, and *P* = 0.17 for the interaction effect). The effect of TNC seemed most pronounced at 6 h after infection.

In contrast, the absence of TNC had no influence on BALF neutrophil content (Fig. 3B, *P* = 0.83), or on the extent of neutrophil CD11b expression (Fig. 3C, *P* = 0.84). To further study neutrophil activity, we measured the neutrophil products myeloperoxidase (Fig. 3D) and neutrophil elastase (Fig. 3E) in BALF. While both proteins were highly present after infection, neither was dependent on the presence of TNC (*P* = 0.96 and *P* = 0.66 for myeloperoxidase and neutrophil elastase, respectively).

### TNC protects against early tissue pathology during infection with MRSA.

**FIG 4:**
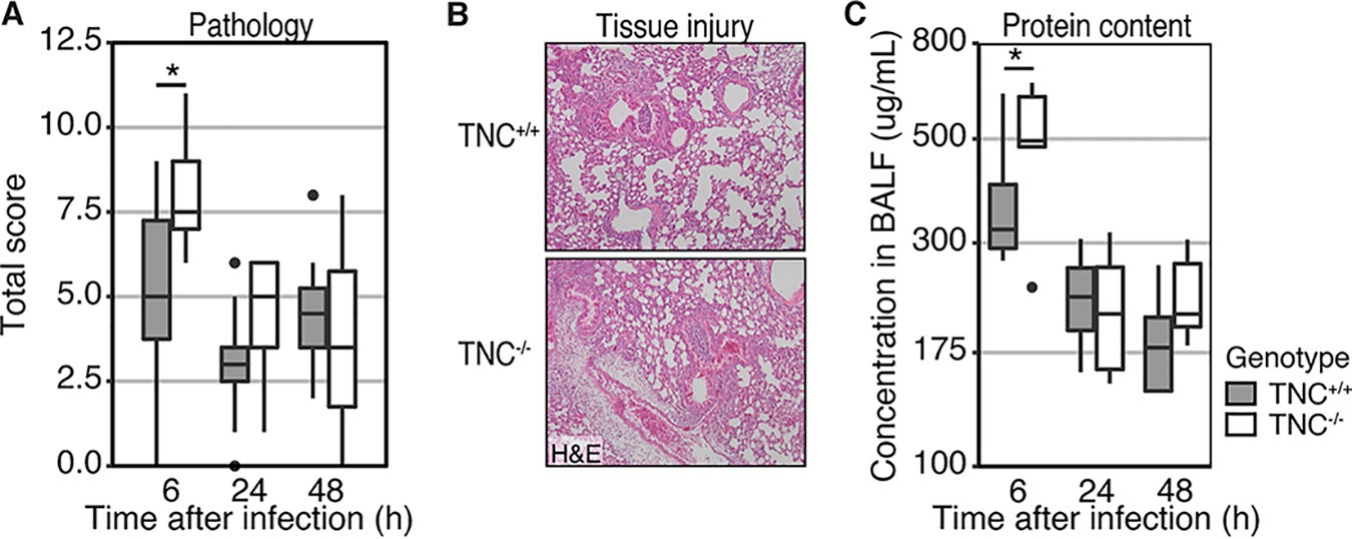
Tenascin C offers limited protection against early tissue pathology during methicillin-resistant Staphylococcus aureus-induced pneumonia. Mice were infected through intranasal administration of MRSA. Mice were sacrificed at 6, 24, and 48 h thereafter and lung tissue and BALF were collected. (A) To determine total tissue pathology, tissue was fixed and embedded in paraffin. H&E-stained slides were prepared and subsequently scored for tissue pathology by a pathologist blinded for the group condition. (B) Representative H&E-stained slides of TNC^+/+^ (top) and TNC^−/−^ mice (bottom) at 6 h after infection. (C) As a measure for integrity of the pulmonary barrier, protein leak into the alveolar space was measured as total protein content of the BALF trough a bicinchoninic acid protein assay. Data are shown as Tukey boxplots. Statistical analysis was performed using linear models. *, *P* < 0.05.

MRSA infections are known to cause severe tissue injury that can persevere beyond the initial inflammation (24). As TNC plays an important role during tissue injury and repair, we studied the development of tissue pathology during the acute and late phase of MRSA-induced pneumonia. Across the model, TNC^−/−^ mice showed more severe tissue pathology compared to TNC^+/+^ mice, as determined by the semi-quantitative scoring system described in the Materials and Methods section (*P* = 0.01 for genotype and *P* = 0.01 for the interaction term, Fig. 4A and B). At 6 h after infection, TNC^−/−^ mice showed significantly more severe tissue pathology compared to TNC^+/+^ mice (*P* = 0.013). After 24 h, though, tissue pathology decreased to a similar level in both genotypes (*P* = 0.26), which persisted until 48 h after infection (*P* = 0.60).

A decrease in the integrity of the pulmonary vascular and epithelial barriers can be observed through the general leakage of proteins into the alveolar space. We therefore quantified total protein content in BALF (Fig. 4C). In the absence of TNC, total protein content was increased (*P* = 0.03). Specifically, TNC^−/−^ showed more protein leakage during acute infection (*P* = 0.02). After 24 h, protein leakage had normalized (*P* = 0.70), although a trend remained at 48 h (*P* = 0.08). This suggests that TNC plays a protective role against tissue injury during acute MRSA-induced pneumonia.

## DISCUSSION

TNC has been reported as a putative damage-associated molecular pattern molecule released during tissue damage that promotes and sustains inflammation (3, 4, 25). Beyond its proinflammatory properties, TNC plays an important role during tissue remodeling and repair, including in models of lung inflammation and fibrosis (1, 11–13). However, both the form and function of TNC can be highly affected by the (patho)biological context in which it is produced (26). Here, we investigated the role of TNC during MRSA-induced pneumonia, which is known to be associated with extensive damage to pulmonary tissue (22). We hypothesized that TNC would provide a positive feedback loop for inflammation and that TNC^−/−^ mice would be relatively protected from excessive lung inflammation and injury. In contrast to our hypothesis, TNC had a limited role in the host response to MRSA in the airways. TNC deficiency even was associated with more lung inflammation and injury early after MRSA infection, as indicated by higher pathology scores and increased protein leak into the bronchoalveolar compartment.

TNC mRNA was induced in the lung transiently during MRSA-induced pneumonia, with a strong induction at 6 h after infection, decreasing thereafter. In contrast, TNC protein expression was detected in the lung tissue particularly at 24 and 48 h after infection. This delay is in line with literature reporting that TNC protein levels depend on *de novo* synthesis, which includes elaborate post-transcriptional modifications, followed by release into the ECM taking place after approximately 24 h (25), as well as with our earlier observations during Klebsiella Pneumoniae-induced pneumosepsis (27).

Previous reports have shown that TNC mediates immune responses of monocytes and macrophages (3, 4, 8, 25, 28). In the absence of TNC, macrophage TNF-α production in response to Toll-like receptor 4 (TLR4) stimulation was severely impaired due to changes in miR-155 signaling (25). In contrast, in this study the absence of TNC did not affect the infiltration or activation of monocytes and macrophages in the lung. We did observe increased levels of IL-6 in the TNC^−/−^ group, which contrasts with earlier studies where TNC has been described as especially potent in eliciting an IL-6 response (28). Notably, while previous investigations on the role of TNC primarily focused on TLR4 activation through lipopolysaccharide (3, 4, 13, 25, 28) or TLR3 activation through Poly(I:C) or viral mediators (29, 30), MRSA is predominantly recognized through TLR2 (31). Further studies are needed to investigate the relationship between immunomodulation by TNC and different TLR signaling pathways.

Further complicating the matter, TNC itself can activate macrophages in a TLR4-dependent manner, inducing cellular responses that are partially similar (including induction of cytokine production) and partially distinct from the LPS-TLR4 interaction (28). Additionally, in certain inflammatory contexts, TNC can play an immunosuppressive role in which different isoforms might be involved (32). Future work should therefore aim to differentiate between TNC-miR-155-dependent macrophage responses and the role of autologous TNC-TLR4 signaling in the context of lung infection and injury. The current study indicates TNC may have a different effect in the context of a TLR2-mediated immune response, and again highlights the context-dependent nature of TNC biology.

Neutrophils are a key player in the clearance of MRSA, as well as main drivers of collateral tissue damage (22). While neutrophils do not express TNC upon activation, TNC plays a role in neutrophil chemotaxis (33, 34). In a model of spinal cord surgery, the absence of TNC leads to an increased neutrophil infiltration at the site of injury (33). In contrast, another investigation reported an inhibitory effect of TNC on neutrophil chemotaxis *in vitro* (35). In the current study, we found that in the absence of TNC, neutrophil recruitment into lung tissue was somewhat increased at 6 h after infection, compared to the TNC-sufficient condition. At later time points, this difference was no longer present due to a more delayed rise in neutrophil counts in TNC-sufficient lungs. Remarkably, TNC deficiency did not impact neutrophil influx into the bronchoalveolar space, suggesting that TNC may affect neutrophil migration through tissue, while leaving trans-endothelial and trans-epithelial migration toward the bronchoalveolar space unaffected. One might speculate that constitutively present TNC in the ECM may hamper neutrophil migration through tissue. Clearly, future studies are needed to elucidate the mechanisms by which TNC affects neutrophil migration in the lungs.

In contrast to our earlier observations in a model of pneumosepsis (27), we did not observe any differences in bacterial outgrowth or immune activation during MRSA-induced pneumonia. However, we did again observe an increase in tissue pathology and protein leak into the bronchoalveolar space in the absence of TNC early after infection, suggesting the integrity of the pulmonary barrier was compromised. This suggests that, in addition to its role in wound healing and tissue remodeling, TNC also protects tissue from injury during the initial insult. After 24 h, we did not observe any differences between the genotypes, which could be due to other mediators becoming more important over time and/or the decreasing tissue pathology at the later time points.

The current study is the first to investigate the functional role of TNC during Gram-positive pneumonia. Contrary to previous studies of sterile inflammation, but in line with our previous study of bacterial pneumonia, we found only modest alterations in the host response in the absence of TNC. Future studies may aim to confirm this observation in a model mimicking enhanced induction of TNC during a bacterial challenge, instead of the TNC^−/−^ context describe here. Nonetheless, in contrast to our expectations, these data argue against an important immunoregulatory role for TNC in MRSA-induced pneumonia.

## MATERIALS AND METHODS

### Ethical statement.

The experiments were reviewed and approved by the Central Authority for Scientific Procedures on Animals (CCD) and the Animal Welfare Body (IvD) of the Academic Medical Center (AMC), University of Amsterdam (identification number DIX288-BP-1). The animal care and use protocol adhered to the Dutch Experiments on Animals Act (WOD) and European Directive of 22 September 2010 (Directive 2010/63/EU) in addition to the Directive of 6 May 2009 (Directive 2009/41/EC).

### Animals.

TNC^+/+^ and TNC^−/−^ mice in a C57BL/6 background (36) were bred in parallel from heterozygous parents. Mice were housed in individually ventilated cages enriched with disposable homes and nesting paper, and provided with food and water *ad libitum*. All mice were bred and housed at the Animal Research Institute AMC under specific pathogen-free conditions. Mice were acclimatized in the procedure room for at least 1 week before commencement of the experiment. Mice entered experiments at 8 to 10 weeks of age and in good health. Both genders were used for experiments and groups were sex matched. Mice were assessed on their welfare (including posture and activity) throughout their stay at the facility.

### Study design.

Experimental groups consisted of 8 mice per time point and per genotype. Home cages were placed in a random order, which was then used throughout the experiment. Pneumonia was induced by intranasal administration of 10^7^ CFU of MRSA USA 300 (BK11540) in 50 μl of sterile isotonic saline, as previously described (37). Inoculation was performed under light sedation with 2 to 3% isoflurane in 100% O_2_ to ensure calm inhalation of the inoculum. Mice were euthanized at 6, 24, or 48 h after inoculation by intraperitoneal injection of ketamine (125 mg/kg) and dexmedetomidine (300 μg/kg), followed by cardiac puncture. Blood was collected into heparin tubes (Microtainer, BD Biosciences, NJ). Unilateral bronchoalveolar lavage fluid (BALF) was collected as described below. The contralateral lung, as well as the spleen and liver, were harvested and homogenized in sterile saline (weight:volume 1:5) using a tissue homogenizer (Biospec Products, Bastlesville, OK). CFU in organ homogenates and blood were determined from serial dilutions plated on blood agar plates incubated at 37°C for 14 h (38).

### Bronchoalveolar lavage.

BALF was collected as described previously (37). In brief, the trachea and bronchi were exposed through a midline incision. The left main bronchus was ligated and the trachea was cannulated with a sterile 22-gauge Abbocath-T catheter (Abbott Laboratories, Sligo, Ireland). Unilateral, right-sided BAL was performed by instilling three 0.3-ml aliquots of sterile phosphate-buffered saline (PBS) and 0.7 to 0.9 ml of BAL fluid was retrieved per mouse.

### Flow cytometry.

Cells in BALF were stained with fixable viability dye and anti-murine CD45-PE Fluor61 (eBioscience, San Diego, CA), SiglecF-Alexa647, CD11b-PE Cy7 (BD Bioscience, Franklin Lakes, NJ), CD11c-PerCP Cy5.5, Ly-6C AlexaFluor700 (BD Pharmingen, San Jose, CA), and Ly-6G-FITC (Biolegend, San Diego, CA). Cells were fixed and red blood cells were removed using fix/lyse solution according to manufacturer’s instruction (eBioscience, San Diego, CA). Quantification of total cell content was performed by addition of precision count beads (Biolegend, San Diego, CA) before measurement. Expression levels of activation markers were quantified via the median fluorescent intensity (MFI). Data were collected on a Canto II flow cytometer (BD Bioscience, Franklin Lakes, NJ) and analyzed using FlowJo software v10.7.1 (Treestar, Palo Alto, CA, USA). The full gating strategy can be found in Fig. S1.

### Assays.

BALF supernatants were stored at –20°C until analysis. Tumor necrosis factor (TNF)-α (Thermo Fisher Scientific, Waltham, MA), interleukin (IL)-1β, IL-6, myeloperoxidase (MPO), and neutrophil elastase (all R&D Systems, Minneapolis, MN) were measured by enzyme-linked immunosorbent assay (ELISA) according to the manufacturer’s instructions. Total protein content in BALF was measured with the Pierce BCA protein assay kit (Thermo Fisher Scientific, Waltham, MA) according to the manufacturer’s instructions.

### Histopathology.

Lungs were collected and fixed in 10% formalin in PBS for at least 16 h, transferred to 70% ethanol, and embedded in paraffin. Four-micrometer paraffin-embedded lung sections were stained with hematoxylin and eosin (H&E). Slides were coded and lung inflammation and damage were scored by a pathologist blinded for group identity. To score lung inflammation and damage, the entire lung surface was analyzed with respect to the following parameters: interstitial inflammation, edema, endothelialitis, bronchitis, and pleuritis. Each parameter was graded on a scale of 0 to 4 (0: absent; 1: mild; 2: moderate; 3: severe; 4: very severe), as visualized in reference 27. The percentage pneumonia was scored and graded according on a scale of 0 to 4 (0: absent; 1: 5 to 20% confluent pneumonia; 2: 21 to 40%; 3: 41 to 60%; 4: 61 to 80%; 5: 81 to 100%). Finally, the number of thrombi was counted. The pathology score was expressed as the sum of the scores for each parameter as previously described (39) and visualized (27). Slides were photographed with a microscope equipped with a digital camera (Leica CTR500).

### Immunohistochemistry.

To visualize TNC protein in the lung, paraffin-embedded lung sections were unmasked by boiling in a 10 mM pH 6 citrate solution. Slides were blocked with 5% normal goat serum in PBS and incubated with the rabbit polyclonal primary anti-TNC antibody (1:100, AB19011, EMD Millipore, Temecula, CA). Slides were washed with 0.1% Triton X-100 in PBS (Sigma-Aldrich, St. Louis, MO) followed by 1:200 diluted secondary anti-rabbit biotinylated antibody (Jackson ImmunoResearch, West Grove, PA). Slides were washed with 0.1% Triton X-100 in PBS and treated with peroxide solution (0.6% H_2_O_2_ [Sigma-Aldrich, St. Louis, MO] in methanol). Slides were washed with 0.1% Triton X-100 in PBS and then were incubated with 3,3 ′-diaminobenzidine developing solution (Vector Lab, Burlingame, CA) for 1 h at room temperature. The signal detection was done with the Elite ABC system (Vectastain, Burlingame, CA) and hematoxylin staining was performed. The sections were embedded into ProLong Gold antifade reagent (Invitrogen, Waltham, MA). Sections were examined using an Axio Imager A1. Pictures were taken with an AxioCam Icc3 camera and Axiovision software (all from Zeiss, Jena, Germany).

Neutrophil influx was determined through immunohistochemical staining with anti-Ly-6G-FITC antibody (1A8, Biolegend, San Diego, CA) as described previously (40). Quantification was performed by digital image analysis. Slides were scanned with the Philips IntelliSite Ultra Fast Scanner 1.6RA (Phillips Digital Pathology Solutions, Best, The Netherlands) and the amount of immune positivity was measured as percentage of the total lung surface area using Fiji on ImageJ 1.53c (41, 42).

### Quantitative PCR.

To determine pulmonary TNC mRNA expression, RNA was isolated from homogenized lung tissue using the Nucleospin RNA isolation kit according to the manufacturer’s protocol (Macherey-Nagel, Düren, Germany). cDNA was synthesized using M-MLV reverse transcriptase with oligo(dT) primers according to the manufacturer’s protocol (Promega, Madigon, WI). TNC and HPRT1 mRNA levels were determined through quantitative PCR using the SensiFast No-ROX kit (Bioline, London, UK) measured on a LightCycler 480 (Roche, Basel, Switzerland) using the following primers: mTNC forward: GATGCCAAGAGTCGCTACAA; mTNC reverse: TGTGTCCTTGTCATAGGTGGA; mHPRT1 forward: AGTCAAGGGCATATCCAACA; mHPRT1 reverse: CAAACTTTGCTTTCCGGGT. The data was analyzed using LinReg PCR 2020.0 (43). TNC mRNA expression is presented after normalization by HPRT1.

### Statistical analysis.

Statistical analysis was performed in R 3.6.3 (44). Figures were created with ggplot2 3.3.0 (45). Non-normal data was normalized through log_10_ transformation for analysis, and is presented back-transformed through their antilog. Datapoints that were below the lower limit of detection were imputed on the lower limit of detection. In figures, data is shown as Tukey box-and-whisker plots. Criteria of normal distribution and homogeneity of variance, examined with Shapiro’s or Levene’s tests respectively, were acceptable (*P* > 0.01). Two-group comparisons were made using independent *t* tests. Differences between groups over time were tested using linear models, with time and genotype treated as interaction variable, and tested by ANOVA F statistics. Interaction terms are reported only when *P* < 0.1. Post-hoc analysis was performed in comparisons with a *P* < 0.05 for genotype or a *P* < 0.01 for the interaction term. Time was dummy coded with the first measurement serving as reference. A *P* value of <0.05 was considered statistically significant.
