# Supplementary material for: Tenascin C Has a Modest Protective Effect on Acute Lung Pathology during Methicillin-Resistant Staphylococcus aureus-Induced Pneumonia in Mice
Source: Microbiol Spectr. 2021 Jul 28;9(1):10.1128/spectrum.00207-21. doi: 10.1128/spectrum.00207-21 (PMC8552697; doi:10.1128/spectrum.00207-21)
Supplement: SUPPLEMENTAL FILE 1 — Supplemental material. Download SPECTRUM00207-21_Supp_1_seq6.pdf, PDF file, 0.2 MB [file spectrum00207-21_supp_1_seq6.pdf]

Supplemental Figure 1: FACS gating strategy

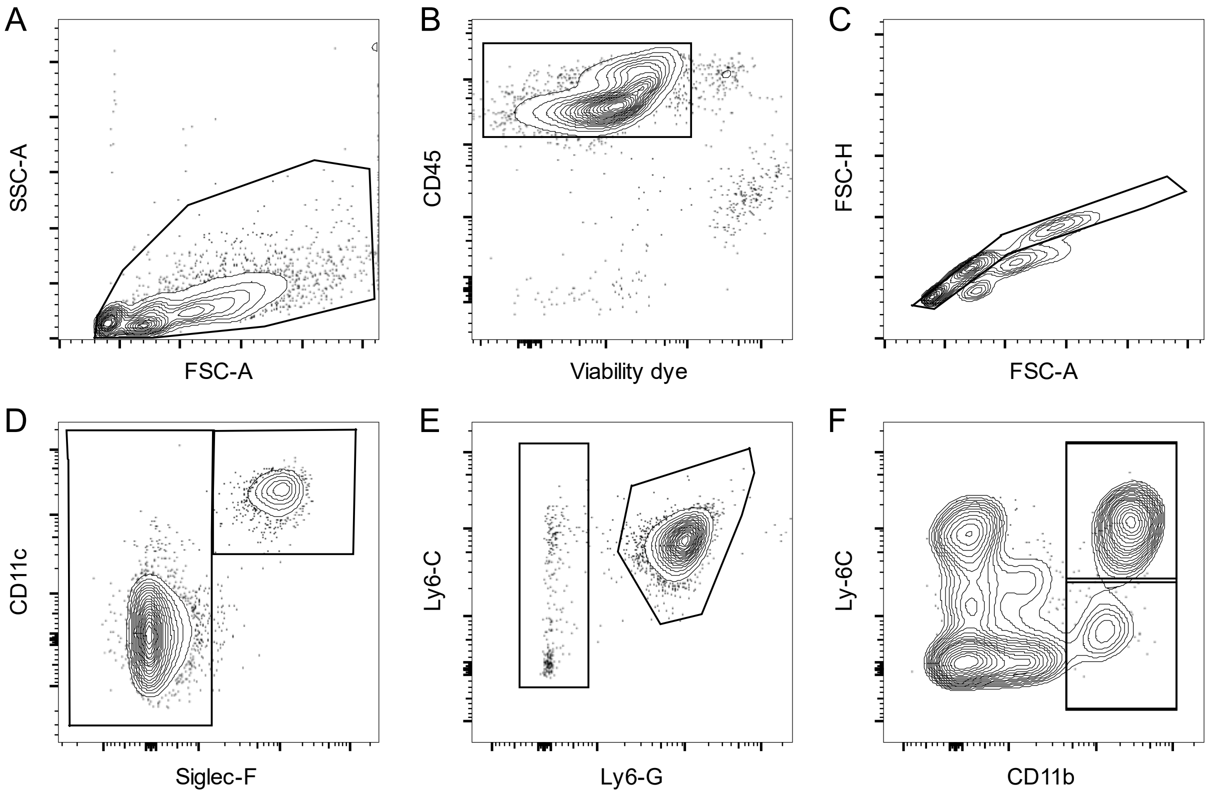

BALF was collected and prepared for FACS analysis. **A**) Total cell content was selected based on scatter. **B**) $CD45^+$viabilitydye$^-$ cells were selected as viable leukocytes. **C**) Duplets were removed and the remaining population was defined as total leukocyte count. Within this subset, **D**) $CD45^+CD11c^+$Siglec-F^+ cells were identified as alveolar macrophages. **E**) Within the $CD45^+$ $CD11c^-$ Siglec-F^- population, Ly-$6G^+$Ly-$6C^+$ cells were identified as neutrophils. **F**) The remaining cells were identified as monocytes based on the presence of CD11b. $CD11b^+Ly6C^{hi}$ cells were determined as inflammatory monocytes, whereas $CD11b^+Ly6C^{lo}$ cells represent the non-inflammatory monocytes. The figure shows a representative sample, as 5% contour plot with outliers.

Supplemental table 1: Number of cultures positive for Staphylococcus aureus

	Spleen						Liver						Blood					
	6		24		48		6		24		48		6		24		48	
	+	-	+	-	+	-	+	-	+	-	+	-	+	-	+	-	+	-
$TNC^{+/+}$	3	5	4	4	1	7	4	4	3	5	0	8	0	8	4	4	1	7
$TNC^{-/-}$	6	2	3	5	3	5	1	7	2	6	0	8	0	8	2	6	2	6

Mice were intranasally infected with S. aureus. Upon sacrifice at 6, 24 or 48 hours after infection, blood was collected as well as spleen and liver tissue, which were homogenized in sterile saline. To determine the bacterial load, all samples were plated on blood agar plate and incubated overnight. The table displays the N of cultures that were found to be positive (+) or negative (-) for Staphylococcus aureus, in both the TNC sufficient ($TNC^{+/+}$) and the TNC deficient ($TNC^{-/-}$) group.
